# A Review and Comparative Analysis of Transoral Surgical Treatment versus Conservative Management in Early-Stage Oropharyngeal Cancer

**DOI:** 10.3390/jpm14030283

**Published:** 2024-03-04

**Authors:** Simona Andreea Rujan, Serban Vifor Gabriel Bertesteanu, Raluca Grigore, Bogdan Popescu, Mihnea Condeescu-Cojocarita, Nicolaescu Alexandru, Gloria Simona Bertesteanu, Teodora Elena Schipor-Diaconu, Anca Ionela Cirstea, Mihai Dumitru Tudosie, Irina-Doinita Popescu, Bianca Petra Taher

**Affiliations:** 1 Department 12—Otorhynolaryngology, Ophthalmology, Faculty of Medicine, “Carol Davila” University of Medicine and Pharmacy, 050474 Bucharest, Romania; serban.bertesteanu@umfcd.ro (S.V.G.B.); raluca.grigore@umfcd.ro (R.G.); bogdan_popescu@umfcd.ro (B.P.); mihnea.condeescu@gmail.com (M.C.-C.); alexandru.nicolaescu@umfcd.ro (N.A.); simona-gloria.munteanu@drd.umfcd.ro (G.S.B.); elena-teodora.diaconu@drd.umfcd.ro (T.E.S.-D.); anca-ionela.cirstea@drd.umfcd.ro (A.I.C.); mihai-dumitru.tudosie@drd.umfcd.ro (M.D.T.); irina-doinita.oana@drd.umfcd.ro (I.-D.P.); 2 Department of ENT, Head and Neck Surgery, Colţea Clinical Hospital, 030167 Bucharest, Romania; 3 “Prof. Dr. Dimitrie Gerota” Emergency Hospital, 030167 Bucharest, Romania; 4 Central Military Emergency Hospital “Carol Davila”, 010825 Bucharest, Romania

**Keywords:** neoplasm, oropharynx, transoral, microsurgery

## Abstract

Introduction: Oropharyngeal cancer requires a comprehensive evaluation of treatment options, including surgery, radiation therapy, and chemotherapy. It is crucial to customize these treatments based on the stage of the tumor and the overall health of the patient, enabling personalized or combined approaches. Transoral surgical techniques have regained popularity due to the advancements and limitations of non-surgical strategies. The potential influence of surgical procedures on patients’ quality of life highlights the need for careful intervention selection; among them, the transoral approach has proven to be especially beneficial for early-stage oropharyngeal neoplasms. Methods: To explore potential treatments for early-stage oropharyngeal malignancies, this study carefully reviews the literature, using information from papers, current research, and global databases. The review protocol commenced on November 2023. A comprehensive search of the PUBMED database was undertaken, employing pertinent terms associated with oropharyngeal, transoral surgery or radiotherapy, robotic surgery, and chemotherapy. Results: Treating early-stage oropharyngeal neoplasms is particularly intriguing due to the multitude of variables influencing treatment decisions, leading to ongoing debates in specialized literature. Regardless of the chosen approach, maintaining a high quality of life is crucial. To assess this, standardized questionnaires from the European Organization for Research and Treatment of Cancer were employed, revealing superior outcomes for patients solely undergoing surgical intervention. Additionally, in the realm of specialized literature, cases of HPV-positive oropharyngeal neoplasms are recognized for their heightened radiosensitivity and more favorable long-term prognosis. Conclusions: Surgical intervention and radiotherapy are the main treatment options for oropharyngeal cancer, and they can be used separately or together for maximum effectiveness. Amid ongoing discussions, determining the superior effectiveness between the two options continues to be a matter of debate. This study provides a comprehensive analysis, offering valuable perspectives for future discussions. Neoplasm in the oropharynx can be effectively treated using transoral microsurgery.

## 1. Introduction

The oropharynx is located in the middle of the pharynx and stretches from the soft palate to the upper part of the hyoid bone. It consists of a variety of structures, such as the tonsils, soft palate, tongue base, posterior one-third of the tongue, and posterior and lateral pharyngeal walls. The most common type of oropharyngeal cancers is squamous cell carcinoma (OPSCC), which develops from the squamous non-keratinizing epithelial cell lining of the oropharynx. It makes up about 90% of all cases [1].

Oropharyngeal carcinoma can be classified into two main groups from a therapeutic standpoint: HPV-associated, which is caused by an oral human papillomavirus infection, and non-HPV-associated, which is primarily linked to tobacco smoking and alcohol consumption. Several risk factors that contribute to the development of certain conditions include genetic mutations, betel quid chewing, poor nutrition, marijuana smoking, and exposure to asbestos. According to research in specialized literature, patients with HPV-associated OPSCC tend to be younger and have a higher likelihood of being male compared to those not affected by HPV infection [2,3,4].

**Figure 1:**
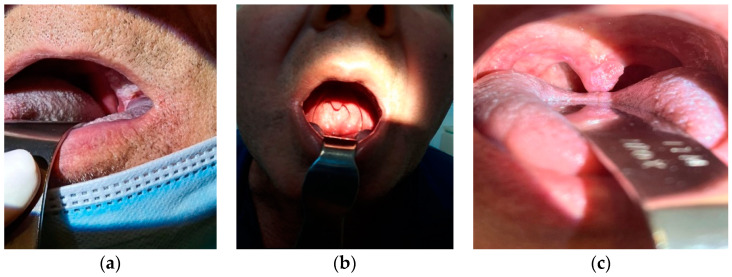
(**a**–**c**) Clinical presentation of different patients with oropharyngeal cancer from Coltea Clinical Hospital.

The clinical presentation can be easily modified based on the location, as shown in Figure 1a–c. Common symptoms of oropharyngeal neoplasms include pain, difficulty swallowing, and weight loss, regardless of where they are located. In addition, if the neoplasm is located at the tonsils or the base of the tongue, it may also cause ear pain on the same side, known as ipsilateral otalgia.

Since 2018, according to AJCC, staging has been modified according to HPV status (Table 1 and Table 2).

HPV infection adds a level of variability to the staging process. It is worth mentioning that in patients with HPV-positive stage II, there are associations with T3,4 and N2, which is different from the non-HPV category where stage II only includes T2 and N0. This is a result of the better outcomes of larger locally extended tumors of HPV-positive OPSCC than those of similar size but HPV-negative.

Possible diagnoses include conditions like lichen planus, oral candidosis, traumatic lesions, or actinic keratosis. There is a wide range of treatment options available for oropharyngeal cancer, such as surgery, radiotherapy, and chemotherapy. The choice of these modalities, whether used alone or in combination, depends on factors such as the stage of the tumor and the patient’s overall performance status (ECOG score). In recent years, radiation therapy, either alone or combined with chemotherapy, has become increasingly important in the treatment of oropharyngeal cancer in both Europe and the USA. Highlighting the importance of preserving essential oropharyngeal functions, these centers typically prioritize non-surgical treatments when radiation and chemotherapy are not effective [5].

**Scheme 1:**
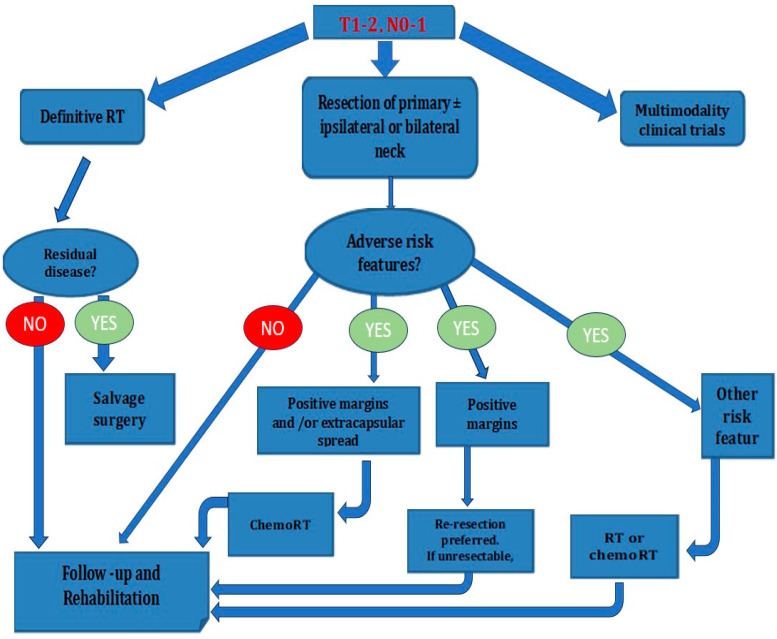
Oropharyngeal cancer treatment.

The standard-of-care is to discuss all cases in a multidisciplinary tumor board for a thorough analysis and decision-making process regarding the best treatment for each patient (Scheme 1).

Regarding surgical treatment options in OPSCC, there are two methods for removing the primary tumor: an open approach or a transoral approach. The decision is carefully made by the treating surgeon(s), taking into account their expertise and the resources available.

The transoral approach is typically limited to treating small, easily reachable lesions. Nevertheless, it is crucial to consider the possible drawbacks, including the potential risk of injury to the internal carotid artery and subsequent massive life-threatening bleeding as well as a high possibility of recurrence because of incomplete resection (positive margins) due to poor visualization and limited access. This is especially important given the significant occurrence of cervical metastases, even in the early stages of T1-T2 lesions [6].

This review aims to provide a thorough analysis of the current literature on the comparison between Transoral Surgical Treatment and Conservative Management in Early-Stage Oropharyngeal Cancer.

**Figure 2:**
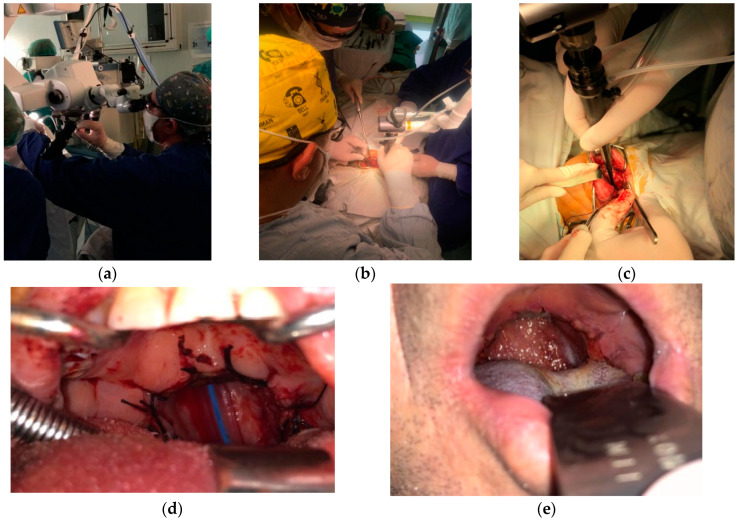
(**a**–**e**) Intraoperative and postoperative views from Laser CO_2_ transoral approach.

Transoral surgery (TOS) is gaining recognition as a safe and effective method for treating upper aerodigestive tract cancer. Numerous studies [7,8,9,10] have demonstrated its positive impact on both functional and oncologic outcomes (refer to Figure 2d,e, which depict day 1 and day 7 after transoral buccopharyngectomy at ENT Coltea Hospital). In addition, it has been found to be associated with shorter hospital stays, improved patient outcomes, and lower rates of surgical complications [8,11].

Transoral endoscopic head and neck surgery (eHNS) represents an innovative technique to minimally invasive oncological procedures in the oropharynx. This technique utilizes carbon dioxide laser microsurgery, specifically transoral laser microsurgery (TLM) (see Figure 2a–c for intraoperative visuals at ENT Coltea Hospital), and/or robotic surgery, known as transoral robotic surgery (TORS).

Transoral surgical techniques have gained renewed interest due to recent technological advancements and the high rate of adverse effects associated with non-surgical treatments. When considering the impact of surgical interventions on quality of life, the transoral surgical approach stands out as a preferred option, particularly for early-stage oropharyngeal neoplasms. Transoral surgical treatment can lead to various complications, including trismus, changes in taste, aspiration, dysphagia, infections, or bleeding. It is worth mentioning that experiencing significant post-transoral surgical bleeding is an uncommon occurrence [9,10,11].

It is recommended that patients who have positive surgical margins and/or extracapsular spread undergo chemoradiotherapy (chemoRT) after their surgery. If the only concern is positive surgical margins, it is recommended to opt for re-resection [11].

If the tumor is considered inoperable, the suggested course of action would be radiotherapy (RT) or chemoRT. The recommended radiation therapy dose usually falls within the range of 66–70 Gy. Complications related to radiotherapy are extensively documented, including mucositis, fibrosis, xerostomia, dermatitis, osteoradionecrosis, neutropenia, and dysphagia. Dysphagia, in particular, is a significant problem in both the short-term and long-term aftermath of radiotherapy, with severe cases sometimes requiring a feeding gastrostomy [10,11].

When faced with a difficult prognosis, patients often struggle with the development of psychiatric disorders. They face challenges in accepting their diagnosis, adjusting to changes in their physical appearance, and considering the possibility of undergoing complex and disfiguring surgeries, as well as demanding cancer treatments. People facing this intricate situation often encounter biases and obstacles when trying to reintegrate into society. Thus, transoral surgical techniques are regarded as more practical and well-received by patients in comparison to traditional treatment methods, provided there is a clear indication for their use in this particular context [11].

When it comes to early-stage oropharyngeal neoplasms, choosing the right treatment is crucial. It requires careful consideration, with a strong emphasis on improving the patient’s quality of life after treatment. This holds true regardless of whether the chosen approach involves surgery using a transoral method or includes radiotherapy treatment.

## 2. Materials and Methods

This paper conducted a thorough examination of the existing literature, incorporating findings from recent studies, articles, and global databases to investigate potential treatments for early oropharyngeal cancers. We found a significant number of articles during our initial search. The articles selected for this study were written in English and focused on reviews conducted on human subjects. Specifically, the reviews compared the effectiveness of the transoral approach with radiotherapy in treating oropharyngeal cancer, and included follow-up data. The exclusion criteria included articles written in languages other than English, articles with limited access to the full text, and studies that specifically focused on advanced-stage oropharyngeal neoplasms.

The research methodology follows the PRISMA flow diagram and protocol, showcasing the process of selecting articles for further analysis. The review protocol began on 15 November 2023. We conducted a thorough search of the PUBMED database, utilizing relevant terms related to oropharyngeal, transoral surgery or radiotherapy, robotic surgery, and chemotherapy. Afterwards, the search terms were used in both Medline and MDPI databases, resulting in a total of 52,578 results, out of which 388 were duplicates.

Using automation tools, a total of 45,839 articles were eliminated from consideration. Furthermore, an additional 2875 articles were excluded as they were published prior to the year 2000. As a result, a total of 3864 articles were carefully reviewed by examining their titles and abstracts. After this thorough screening process, 3247 articles were determined to be excluded. Out of 617 articles, 526 had limited full-text access, and due to time constraints, it was not possible to reach out to the authors for access. As a result, a total of 91 articles were assessed.

Out of these, 47 were excluded as they focused on locally advanced oropharyngeal tumors, while 15 were excluded due to inadequate specification of the type of surgery. In the end, a total of 29 studies were considered for the review, and 14 of them were cited in the article. After reviewers’ report 8 articles were added.

**Scheme 2:**
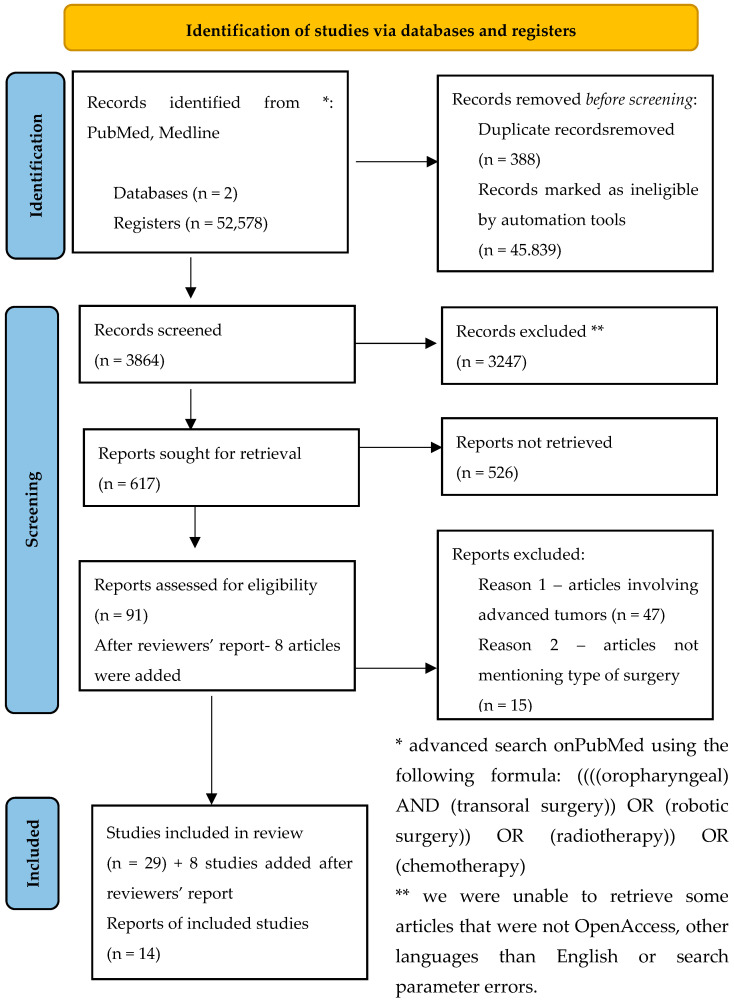
Flow diagram of the research.

The previously mentioned flowchart is depicted in Scheme 2.

## 3. Results

### 3.1. Quality of Life

This investigation thoroughly examined the patients’ quality of life, a crucial aspect under scrutiny. To assess this, meticulous use was made of standardized questionnaires from the European Organisation for Research and Treatment of Cancer (EORTC). In this study, two modules were used: the Head and Neck Cancer Module [12] (EORTC-QLQ-H&N35) and the Core Module [13] (EORTC-QLQ-C30). The selection process and study protocol for patients received approval from the Nuremberg ethics committee. An extensive analysis of long-term results was conducted on 111 eligible patients who had undergone treatment for early-stage oropharyngeal carcinoma. The study conducted a thorough analysis of different treatment options after surgery, including surgery with radiotherapy or chemotherapy. The main emphasis was on assessing the impact of these treatments on the patients’ quality of life. The combined QLQ-C30 and HN35 assessments conducted by the EORTC showcased significantly better outcomes in areas such as social function, speech, nausea, pain, swallowing, dry mouth, financial difficulties, speaking, mouth opening, consistency of saliva, and nutrition for patients who underwent surgical treatment exclusively.

### 3.2. RT versus Surgical Treatment

Psychosocial aspects also showed notable improvements. Typically, the treatment approach for early-stage oropharyngeal squamous cell carcinoma (OPSCC) involves primary surgery or primary radiotherapy (RT). A retrospective study conducted at the Oncological Institute of Lisbon from 2009 to 2014 examined 61 patients with early-stage oropharyngeal neoplasm. The study found that both surgery and primary RT resulted in similar overall survival and relapse-free survival rates. The most common site for surgery was the tonsils, while primary radiation therapy was frequently given at the soft palate and base of the tongue. A study was conducted on OPSCC of the tongue base, which showed a local control rate of 84%. A small number of cases showed a reliance on gastrostomy. Another comprehensive literature study involving 42 articles was conducted to meta-analyze the occurrence of positive safety margins and the factors influencing local tumor control in transoral surgery for oropharyngeal cancers. The findings revealed a 7.8% rate of positive safety margins, which were linked to a lower rate of local tumor control. Positive margins were found to be more likely in T4 stages. Interestingly, the study found that tumor location (tonsilla or tongue base) and the presence of HPV infection did not appear to be predisposing factors for positive safety margins [13,14].

### 3.3. Comparison between Different Types of Transoral Surgery

Numerous studies conducted at individual institutions have found similar results when it comes to treating oropharyngeal squamous cell carcinoma with transoral surgery, using either laser microsurgery or robotic surgery. In a groundbreaking study conducted over 21 years, Canis et al. [15,16] examined the outcomes of 102 patients who underwent transoral laser microsurgery for OPSCC of the tonsil. The results revealed an impressive locoregional control rate of 75% for early tumors. This research holds significant implications for the field and has the potential to make a significant impact on the medical community. In a study involving 30 patients, Weinstein et al. [17,18] found that two years after undergoing primary robotic surgery for oropharyngeal cancer, the overall survival rate was 100% and the locoregional control rate was 97%. A multicenter study involving 410 patients has shown an impressive 91.8% 2-year locoregional control and 94.5% disease-specific survival rates. Significantly, just 50% of the patients necessitated adjuvant radiotherapy (31.3%) or chemoradiotherapy (21.3%). In a 2014 systematic review, the comparison between transoral robotic approaches and intensity-modulated radiotherapy for early OPSCC showed comparable oncologic outcomes at the 2-year mark. Studies conducted by B. Morisod and C. Simon [19] have found that the 5-year overall survival and disease-specific survival rates were similar to those of nonsurgical treatments.

Ultimately, relying solely on surgery to treat early-stage oropharyngeal carcinoma offers significant advantages, especially in terms of functionality, when compared to treatment plans that include additional therapies. Psychosocial aspects also showed notable improvements.

### 3.4. Neck Disection

The early and frequent occurrence of the spread of malignant cells from oropharyngeal squamous cell carcinoma (OPSCC) to the lymph nodes in the neck is well-documented.

In cases of oropharyngeal squamous cell carcinoma (OPSCC), although the existing literature lacks clarity, there appears to be an association between neck dissection and the overall survival of patients diagnosed with early stages of OPSCC [20].

Initially, neck dissections were conducted in a comprehensive manner, such as the Radical Neck Dissection, (dissection from level I to level V with the sacrifice of the sternocleidomastoid muscle, submandibular gland, spinal accessory nerve, and internal jugular vein). Selective Neck Dissection of levels II–IV is the primary method used in modern practice to manage neck therapy for oropharyngeal squamous cell carcinoma [21].

## 4. Discussion

Understanding the importance of acknowledging potential limitations is essential when conducting a literature review. The aim of the review is to provide a thorough compilation of the existing literature on the subject. Decisions regarding the initial stages of oropharyngeal neoplasms require detailed discussions to determine the most effective treatment. However, in the realm of specialized literature, there is a prevailing inclination towards surgical treatment via a transoral approach as opposed to radiotherapy in numerous instances. Many individuals prefer transoral surgical treatment due to its ability to mitigate potential adverse effects associated with radiotherapy. Opting for the latter option can help maintain a higher quality of life for the patient by avoiding certain complications associated with radiotherapy. There has been a noticeable increase in the cases of oropharyngeal squamous cell carcinoma (OSCC), which is believed to be linked to the higher prevalence of human papillomavirus (HPV) infection. In terms of clinical findings, HPV-positive OSCC has been associated with a more positive prognosis and treatment outcome [22,23].

Transoral laser microsurgery (TLM) offers numerous benefits compared to open surgery. These include a quicker recovery period, reduced risk of complications, and enhanced functional outcomes [24,25]. Additionally, preliminary research suggests that TLM shows more favorable results in terms of swallowing function when compared to radiotherapy/chemoradiotherapy (RT/CRT) [26].

The surgical approach also provides the benefit of histopathological characterization of the tumor, which helps in guiding subsequent management and treatment. This advantage is not present with radiotherapy/chemoradiotherapy treatment.

Examining the specialized literature using multi-center studies, it has been shown that transoral robotic surgery is a highly viable option for oropharyngeal squamous carcinoma, resulting in outstanding functional and oncological results. When it comes to treating oropharyngeal neoplasms, TORS stands out for its exceptional functional outcomes, as supported by clinical evidence. In addition, TORS is linked to decreased morbidity and treatment expenses, thanks to shorter hospital stays, all while maintaining the same level of effectiveness in cancer treatment.

Therapeutic decision-making for early-stage oropharyngeal neoplasms can often be quite challenging. Choosing the right oncological treatment and surgical technique is of utmost importance, especially for individuals considering surgical intervention. When presenting treatment options, it is crucial to have meaningful discussions with the patient. It is important to provide detailed information about both the advantages and risks associated with the chosen treatment. It is crucial for the patient to have a thorough understanding of the situation before giving informed consent for the proposed treatment.

Presently, a significant concern revolves around the global surge in oropharyngeal squamous cell carcinoma (OPC) cases, attributed to HPV infection.

The divergence in oropharyngeal neoplasms based on the presence of HPV infection is widely acknowledged, resulting in distinct characteristics and prognosis. However, therapeutic options continue to be a subject of debate in the literature, lacking a consensus on the optimal approach. Decisions are typically contingent on various factors such as patient comorbidities, surgeon expertise, and the disease stage. Nonetheless, the paramount consideration remains the monitoring and preservation of the patient’s quality of life [27,28,29].

The disparities between the two categories are evident right from the risk factors, where HPV-positive oropharyngeal neoplasms predominantly affect younger individuals and are not associated with alcohol or smoking. These distinctions persist in their staging, a notable shift initiated in the 8th edition of the AJCC [30].

It is imperative to note that the management of HPV-positive oropharyngeal neoplasms may deviate from that of non-HPV oropharyngeal cancers. Specialized literature underscores the heightened radiosensitivity of the former, coupled with a notably more favorable long-term prognosis in cases where HPV is present. HPV+OPC achieved a remarkable 5-year overall survival rate of 93.9% in the 8th edition of the American Joint Committee on Cancer (AJCC) staging [31].

Presently, an appealing treatment avenue for HPV-positive oropharyngeal cancers involves de-escalated radiation. This approach has shown efficacy in maintaining a high quality of life, coupled with improved healing rates and reduced adverse effects. This trend is substantiated by specialized literature, contributing to its increasing adoption as a reliable treatment modality, particularly in the early stages of HPV-positive oropharyngeal neoplasms [32].

This article conducts a comparative analysis of various treatment modalities applicable to early-stage oropharyngeal neoplasms, particularly focusing on distinct surgical approaches via a transoral method. Nevertheless, the article is not without limitations. The absence of conclusive findings on the addressed subject is one of them. It is noteworthy that the specialized literature continues to engage in extensive debate regarding this topic.

## 5. Conclusions

Surgical intervention and radiotherapy are commonly used as the main treatment options for oropharyngeal carcinomas, either on their own or in combination. The ongoing debate about the superior effectiveness of the two options continues to be a topic of disagreement. Although the scientific community has not definitively established the differences in oncologic outcomes between primary chemoradiation protocols and transoral surgical (TOS) approaches, retrospective analyses indicate a potential advantage for primary surgery. It is worth mentioning that the results of TOS treatment may show a higher level of effectiveness [33].

In the field of specialized literature, it is worth noting that there is a significant occurrence of negative safety margins in relation to stages T1-2 of oropharyngeal carcinoma. This trend is especially noticeable when frozen section margin mapping is used [34,35]. In order to achieve negative tumor margins, the proficiency of surgeons becomes a crucial factor, particularly for those who have received training in centers specialized in robotic surgery [36].

When compared to HPV-negative cases, HPV-positive conditions are more sensitive to chemotherapy and radiation therapy. Recent results from two published clinical trials [37,38] show that replacing cisplatin with cetuximab in an attempt to reduce toxicity results in significantly worse survival rates, with no appreciable differences in acute or delayed toxicity. Consequently, patients with HPV-positive cancer might be good candidates for a reduction in the intensity of their treatment.

The treatment strategies for oropharyngeal squamous cell carcinoma (SCC) are expected to continue evolving within a multidisciplinary framework. This trajectory is in line with a continuous effort to reduce the intensity of therapeutic interventions, focusing on a collective attempt to minimize the negative effects associated with the treatment process. The ever-changing realm of oropharyngeal SCC management showcases a sophisticated comprehension and an unwavering dedication to improving treatment methods. Given that up to 30% of patients with cT1–T2 N0 stage may present with hidden nodal disease, it is recommended that patients undergoing primary surgery have ipsilateral selective neck dissection. For precise pathological staging of midline localisation, contralateral neck dissection may also be considered [39].

## Figures and Tables

**Table 1:** AJCC (8th edition) Prognostic Stage Groups for NON-HPV-Associated (p16-) OPSCC.

T Category	N Category	M Category	Stage Group
Tis	N0	M0	0
T1	N0	M0	I
T2	N0	M0	II
T3	N0	M0	III
T1, T2, T3	N1	M0	III
T4a	N0, N1	M0	IVA
T1, T2, T3, T4a	N2	M0	IVA
Any T	N3	M0	IVB
T4b	Any N	M0	IVB
Any T	Any N	M1	IVC

**Table 2:** AJCC (8th edition) Prognostic Stage Groups for HPV-Associated (p16+) OPSCC.

T Category	N Category	M Category	Stage Group
T0, T1 or T2	N0, N1	M0	I
T0, T1 or T2	N2	M0	II
T3 or T4	N0, N1	M0	II
T3 or T4	N2	M0	III
Any T	Any N	M1	IV

## References

[1] American Cancer Society (2021). American Cancer Society: Cancer Facts and Figures 2021.

[2] Amin M.B., Edge S.B., Greene F.L., Byrd D.R., Brookland R.K., Washington M.K., Gershenwald J.E., Compton C.C., Hess K.R., Sullivan D.C. (2017). HPV-Mediated (p16+) Oropharyngeal Cancer. AJCC Cancer Staging Manual.

[3] Amin M.B., Edge S.B., Greene F.L., Byrd D.R., Brookland R.K., Washington M.K., Gershenwald J.E., Compton C.C., Hess K.R., Sullivan D.C. (2017). Oropharynx (p16-) and Hypopharynx. AJCC Cancer Staging Manual.

[4] Gunn G.B., Debnam J.M., Fuller C.D., Morrison W.H., Frank S.J., Beadle B.M., Sturgis E.M., Glisson B.S., Phan J., Rosenthal D.I. (2013). The impact of radiographic retropharyngeal adenopathy in oropharyngeal cancer. Cancer.

[5] Holsinger F.C., Laccourreye O., Weber R.S. (2005). Surgical approaches for cancer of the oropharynx. Oper. Tech. Otolaryngol.-Head Neck Surg..

[6] Steiner W., Ambrosch P. (2000). Endoscopic Laser Surgery of the Upper Aerodigestive Tract: With Special Emphasis on Cancer Surgery, Chapter 5.

[7] Steiner W., Ambrosch P. (2000). Endoscopic Laser Surgery of the Upper Aerodigestive Tract: With Special Emphasis on Cancer Surgery, Chapter 6.

[8] Ellies M., Steiner W. (2007). Peri- and postoperative complications after laser surgery of tumors of the upper aerodigestive tract. Am. J. Otolaryngol..

[9] Kutter J., Lang F., Monnier P., Pasche P. (2007). Transoral laser surgery for pharyngeal and pharyngolaryngeal carcinomas. Arch. Otolaryngol. Head Neck Surg..

[10] Grant D.G., Salassa J.R., Hinni M.L., Pearson B.W., Hayden R.E., Perry W.C. (2008). Transoral laser microsurgery for recurrent laryngeal and pharyngeal cancer. Otolaryngol. Head Neck Surg..

[11] Salassa J.R., Hinni M.L., Grant D.G., Hayden R.E. (2008). Postoperative bleeding in transoral laser microsurgery for upper aerodigestive tract tumors. Otolaryngol. Head Neck Surg..

[12] Aaronson N.K., Ahmedzai S., Bergman B., Bullinger M., Cull A., Duez N.J., Filiberti A., Flechtner H., Fleishman S.B., De Haes J.C.J.M. (1993). The European Organization for Research and Treatment of Cancer QLQ-C30: A quality-of-life instrument for use in international clinical trials in oncology. J. Natl. Cancer Inst..

[13] Gorphe P., Simon C. (2019). A systematic review and meta-analysis of margins in transoral surgery for oropharyngeal carcinoma. Oral Oncol..

[14] Pedro C., Mira B., Silva P., Netto E., Pocinho R., Mota A., Labareda M., Magalhães M., Esteves S., Santos F. (2017). Surgery vs. primary radiotherapy in early-stage oropharyngeal cancer. Clin. Transl. Radiat. Oncol..

[15] Canis M., Martin A., Kron M., Konstantinou A., Ihler F., Wolff H.A., Matthias C., Steiner W. (2013). Results of transoral laser microsurgery in 102 patients with squamous cell carcinoma of the tonsil. Eur. Arch. Otorhinolaryngal..

[16] Canis M., Ihler F., Wolff H.A., Christiansen H., Matthias C. (2013). Oncologic and functional results after transoral laser microsurgery of tongue base carcinoma. Eur. Arch. Otorhinolaryngal..

[17] Weinstein G.S., Quon H., Newman H.J., Chalian J.A., Malloy K., Lin A., Desai A., Livolsi V.A., Montone K.T., Cohen K.R. (2012). Transoral robotic surgery alone for oropharyngeal cancer: An analysis of local control. Arch. Otolaryngol. Head Neck Surg..

[18] Apostol A., Albulescu N., Iurciuc S., Iurciuc M., Bogdan C., Ivan M.V. (2019). Difference in Cardiac Electrical Vulnerability Between Passive Silicone Steroid Eluting Lead vs. Active Screw-in Lead. Mater. Plast..

[19] de Almeida J.R., Byrd J.K., Wu R., Stucken C.L., Duvvuri U., Goldstein D.P., Miles B.A., Teng M.S., Gupta V., Genden E.M. (2014). A systematic review of transoral robotic surgery and radiotherapy for early oropharynx cancer: A systematic review. Laryngoscope.

[20] Hong L., Sina T.J., Henry P.S., Wendell Y.G., Saral M., Rachel C., Benjamin J.L. (2018). Clinical value of transoral robotic surgery: Nationwide results from the first 5 years of adoption. Laryngoscope.

[21] Meccariello G., Catalano A., Cammaroto G., Iannella G., Vicini C., Hao S.P., De Vito A. (2022). Treatment Options in Early Stage (Stage I and II) of Oropharyngeal Cancer: A Narrative Review. Medicina.

[22] Licitra L., Perrone F., Bossi P., Suardi S., Mariani L., Artusi R., Oggionni M., Rossini C., Cantù G., Squadrelli M. (2006). High-risk human papillomavirus affects prognosis in patients with surgically treated oropharyngeal squamous cell carcinoma. J. Clin. Oncol..

[23] Nichols A.C., Dhaliwal S.S., Palma D.A., Basmaji J., Chapeskie C., Dowthwaite S., Franklin J.H., Fung K., Kwan K., Wehrli B. (2013). Does HPV type affect outcome in oropharyngeal cancer?. J. Otolaryngol. Head Neck Surg..

[24] Mydlarz W.K., Chan J.Y., Richmon J.D. (2014). The role of surgery for HPV-associated head and neck cancer. Oral Oncol..

[25] Sinha P., Hackman T., Nussenbaum B., Wu N., Lewis J.S., Haughey B.H. (2014). Transoral laser microsurgery for oral squamous cell carcinoma: Oncologic outcomes and prognostic factors. Head Neck.

[26] Chen A.M., Daly M.E., Luu Q., Donald P.J., Farwell D.G. (2015). Comparison of functional outcomes and quality of life between transoral surgery and definitive chemoradiotherapy for oropharyngeal cancer. Head Neck.

[27] Rischin D., Young R.J., Fisher R., Fox S.B., Le Q.-T., Peters L.J., Solomon B., Choi J., O’Sullivan B., Kenny L.M. (2010). Prognostic significance of p16INK4A and human papillomavirus in patients with oropharyngeal cancer treated on TROG 02.02 phase III trial. J. Clin. Oncol..

[28] Worden F.P., Kumar B., Lee J.S., Wolf G.T., Cordell K.G., Taylor J.M., Urba S.G., Eisbruch A., Teknos T.N., Chepeha D.B. (2008). Chemoselection as a strategy for organ preservation in advanced oropharynx cancer: Response and survival positively associated with HPV16 copy number. J. Clin. Oncol..

[29] Seiwert T.Y., Melotek J.M., Blair E.A., Stenson K.M., Salama J.K., Witt M.E., Brisson R.J., Chawla A., Dekker A., Lingen M.W. (2016). Final results of a randomized phase 2 trial investigating the addition of cetuximab to induction chemotherapy and accelerated or hyperfractionated chemoradiation for locoregionally advanced head and neck cancer. Int. J. Radiat. Oncol. Biol. Phys..

[30] Zanoni D.K., Patel S.G., Shah J.P. (2019). Changes in the 8th edition of the American Joint Committee on Cancer (AJCC) staging of head and neck cancer: Rationale and implications. Curr. Oncol. Rep..

[31] Yamashita Y., Ikegami T., Hirakawa H., Uehara T., Deng Z., Agena S., Uezato J., Kondo S., Kiyuna A., Maeda H. (2019). Staging and prognosis of oropharyngeal carcinoma according to the 8th edition of the American Joint Committee on Cancer Staging Manual in human papillomavirus infection. Eur. Arch. Otorhinolaryngol..

[32] Choi K.H., Song J.H., Kim Y.S., Moon S.H., Lee J., Oh Y.-T., Oh D., Kim J.H., Kim J.W. (2021). Survey of radiation field and dose in human papillomavirus-positive oropharyngeal cancer: Is de-escalation actually applied in clinical practice?. Radiat. Oncol. J..

[33] Morisod B., Simon C. (2016). Meta-analysis on survival of patients treated with transoral surgery versus radiotherapy for early-stage squamous cell carcinoma of the oropharynx. Head Neck.

[34] Park J.O., Lee Y.S., Joo Y.H., Nam I.-C., Cho K.-J., Kim M.-S. (2012). Can the transoral approach secure a cancer-free deep margin in tonsil cancer?. Oral Oncol..

[35] Hinni M.L., Zarka M.A., Hoxworth J.M. (2013). Margin mapping in transoral surgery for head and neck cancer. Laryngoscope.

[36] Cracchiolo J.R., Baxi S.S., Morris L.G., Ganly I., Patel S.G., Cohen M.A., Roman B.R. (2016). Increase in primary surgical treatment of T1 and T2 oropharyngeal squamous cell carcinoma and rates of adverse pathologic features: National Cancer Data Base. Cancer.

[37] Mehanna H., Robinson M., Hartley A., Kong A., Foran B., Fulton-Lieuw T., Dalby M., Mistry P., Sen M., O’Toole L. (2019). Radiotherapy plus cisplatin or cetuximab in low-risk human papillomavirus-positive oropharyngeal cancer (De-ESCALaTE HPV): An open-label randomised controlled phase 3 trial. Lancet.

[38] Gillison M.L., Trotti A.M., Harris J., Eisbruch A., Harari P.M., Adelstein D.J., Jordan R.C.K., Zhao W., Sturgis E.M., Burtness B. (2019). Radiotherapy plus cetuximab or cisplatin in human papillomavirus-positive oropharyngeal cancer (NRG Oncology RTOG 1016): A randomised, multicentre, non-inferiority trial. Lancet.

[39] Monnier Y., Simon C. (2015). Surgery versus radiotherapy for early oropharyngeal tumors: A never-ending debate. Curr. Treat. Options Oncol..

